# Sequential model for predicting patient adherence in subcutaneous immunotherapy for allergic rhinitis

**DOI:** 10.3389/fphar.2024.1371504

**Published:** 2024-07-19

**Authors:** Yin Li, Yu Xiong, Wenxin Fan, Kai Wang, Qingqing Yu, Liping Si, Patrick van der Smagt, Jun Tang, Nutan Chen

**Affiliations:** ^1^ Department of Otorhinolaryngology, The First People’s Hospital of Foshan, Foshan, China; ^2^ Department of Otorhinolaryngology, The Second Affiliated Hospital of Guizhou University of Traditional Chinese Medicine, Guiyang, China; ^3^ Paul C. Lauterbur Research Center for Biomedical Imaging, Shenzhen Institutes of Advanced Technology, Shenzhen, China; ^4^ Department of Radiology, Zhongshan Hospital, Fudan University, Shanghai, China; ^5^ Faculty of Informatics, ELTE University, Budapest, Hungary; ^6^ Machine Learning Research Lab, Volkswagen Group, Munich, Germany

**Keywords:** allergic rhinitis, allergen immunotherapy, adherence, sequential model, latent variable model

## Abstract

**Objective:**

Subcutaneous Immunotherapy (SCIT) is the long-lasting causal treatment of allergic rhinitis (AR). How to enhance the adherence of patients to maximize the benefit of allergen immunotherapy (AIT) plays a crucial role in the management of AIT. This study aims to leverage novel machine learning models to precisely predict the risk of non-adherence of AR patients and related local symptom scores in 3 years SCIT.

**Methods:**

The research develops and analyzes two models, sequential latent-variable model (SLVM) of Stochastic Latent Actor-Critic (SLAC) and Long Short-Term Memory (LSTM). SLVM is a probabilistic model that captures the dynamics of patient adherence, while LSTM is a type of recurrent neural network designed to handle time-series data by maintaining long-term dependencies. These models were evaluated based on scoring and adherence prediction capabilities.

**Results:**

Excluding the biased samples at the first time step, the predictive adherence accuracy of the SLAC models is from 60% to 72%, and for LSTM models, it is 66%–84%, varying according to the time steps. The range of Root Mean Square Error (RMSE) for SLAC models is between 0.93 and 2.22, while for LSTM models it is between 1.09 and 1.77. Notably, these RMSEs are significantly lower than the random prediction error of 4.55.

**Conclusion:**

We creatively apply sequential models in the long-term management of SCIT with promising accuracy in the prediction of SCIT nonadherence in AR patients. While LSTM outperforms SLAC in adherence prediction, SLAC excels in score prediction for patients undergoing SCIT for AR. The state-action-based SLAC adds flexibility, presenting a novel and effective approach for managing long-term AIT.

## 1 Introduction

Allergic rhinitis (AR) is characterized by allergen-specific IgE-mediated inflammation in upper respiratory inflammation with a prevalence of up to 30% worldwide (Meltzer, 2016). In addition to allergen avoidance as the superior criterion, allergen-specific immunotherapy (AIT) aims to induce specific allergen immune tolerance, consequently achieving a status of clinical symptom remission. The repeatable intake of the specific unmodified or chemically modified allergens (allergoids) was the key to maintaining the symptoms. Among these approaches of AIT, subcutaneous immunotherapy (SCIT), sublingual immunotherapy (SLIT), and lymphatic immunotherapy (LIT) are demonstrated as the mainstream treatments regarding efficacy, safety, and side effects. Compared to the SLIT, SCIT is a clinic-dependent treatment in which the patient accepted an allergen extract injection subcutaneously. It is divided into the initial treatment stage (dose accumulation stage) and the maintenance treatment stage (dose maintenance stage). The World Allergy Organization (WAO) recommends that immunotherapy be maintained for three to 5 years and clinically recommended for at least 2 years. Patient adherence is a critical factor in ensuring long-lasting efficacy and sustaining symptom-relieving effects.

Due to the long duration of SCIT, cumbersome process, slow onset, individual differences in treatment effect, and other factors fundamentally impact the completeness of therapeutics. From the reported studies on AIT, the rate of adherence ranged from around 25% to over 90% (Passalacqua et al., 2013). The World health Organization (WHO) adopted the definition of “adherence” as “the extent to which a person’s behavior, such as taking medication, following a diet, or executing lifestyle changes, corresponds with agreed recommendations from a healthcare provider” (Eduardo, 2003). In recent European Academy of Allergy and Clinical Immunology (EAACI) guidelines, it is highlighted to educate patients on how immunotherapy works and on explaining the importance of compliance to the regular doses for 3 years of treatment (Roberts et al., 2018).

The multiple approaches were introduced into the field of improving adherence and supervising patient outcomes with systematic and technological interventions to prevent incomplete discontinuation of the treatment. The intervention from a clinic in advance running through the whole treatment cycle was approved as an effective approach. In facing the multitude of personalized data from patients, how to precisely identify and assess the risk of upcoming non-adherent behavior, a clinical prediction model is promising in the application.

In healthcare, machine learning, especially sequential models, stands at the forefront of innovation, providing new ways to analyze complex medical data and improve patient treatments. Previous research primarily concentrated on non-sequential prediction methods for adherence (Ruff et al., 2019; Wang et al., 2020; Mousavi et al., 2022; Warren et al., 2022). This approach presents a significant limitation in treatment processes, particularly for immunotherapy that often spans extended periods, such as 3 years. These non-sequential methods tend to predict only the overall outcome, overlooking the intricacies of intermediate time steps. To facilitate earlier intervention, a sequential model capable of making predictions at any given time step would be markedly more beneficial. While some subsequent studies have introduced sequential models (Hsu et al., 2022; Singh et al., 2022; Schleicher et al., 2023), their scope was restricted to predicting adherence alone. Our study enhances this approach by incorporating a state-action model, which can predict both adherence and score/state. This advancement allows for more precise and detailed management of AR patients by allergologists. These models excel in processing and analyzing time-dependent data, making them ideal for predicting patient adherence to treatments like SCIT for AIT. By effectively using sequential data, these algorithms uncover temporal patterns and correlations, leading to more accurate and personalized treatment plans.

In this study, in order to introduce the appropriate prediction model into long-period immunotherapy to customize the management of interventions and incorporate patient feedback, we have selected and evaluated two specific sequential models tailored to this scenario. Our findings demonstrate that these models are not only effective in predicting patient adherence to medical treatments but also invaluable in enhancing treatment strategies, thereby making a significant contribution to patient-centered healthcare.

## 2 Methods

### 2.1 Study design

**FIGURE 1:**
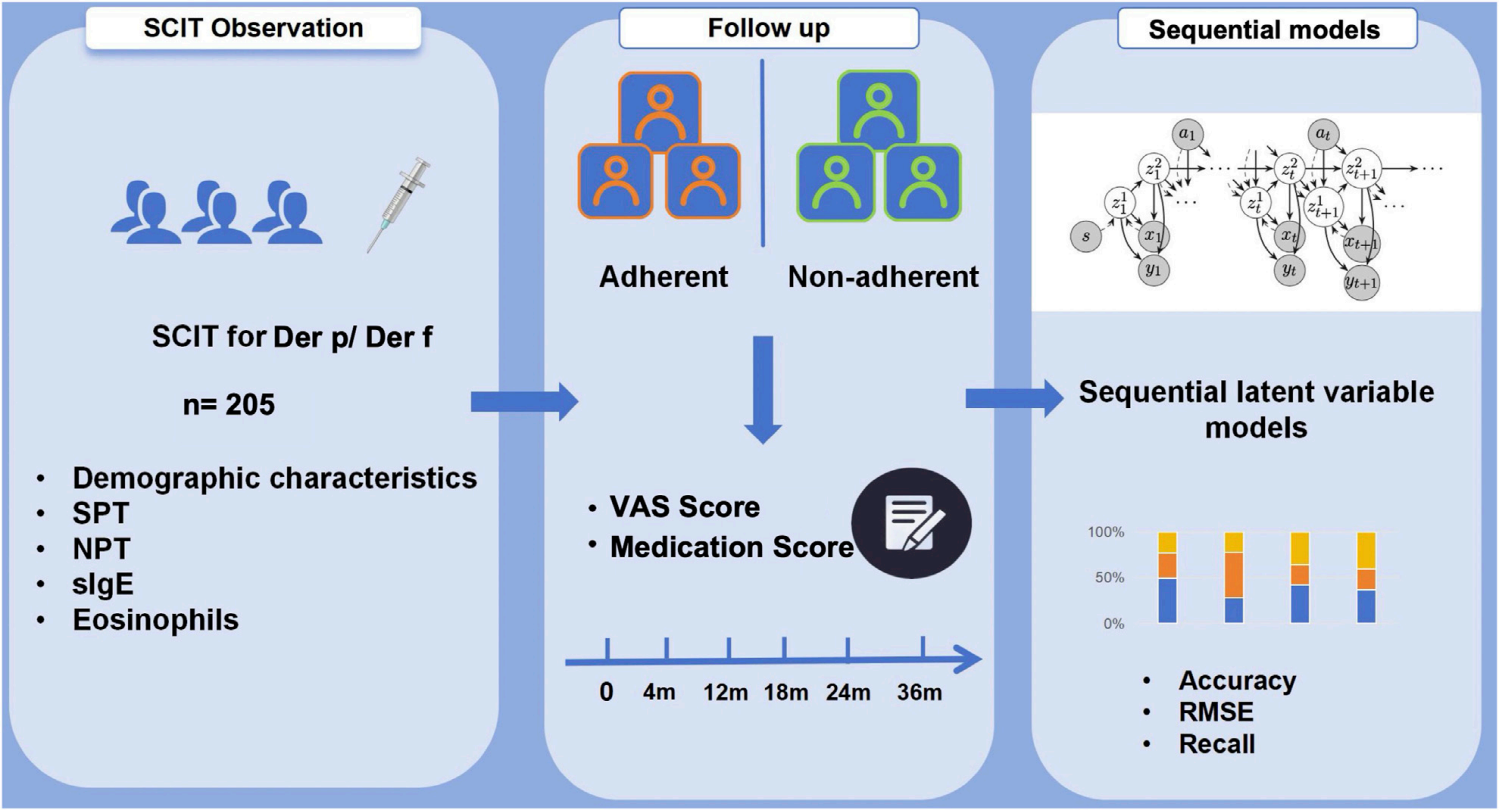
Flowchart on 205 patients treated with SCIT for Der p/Der f allergy during a 36-month treatment period. The adherence was assessed with sequential latent variable models focusing on patient’s demographic characteristics and clinical follow-up data.

The study design is a critical component that shapes the direction and reliability of our research. It includes a systematic approach to selecting the study population, the treatment methods applied, and the evaluation criteria (see Figure 1).

#### 2.1.1 Population

A retrospective analysis including 205 AR patients who started SCIT treatment between August 2018 and September 2019 in the Immunotherapy Center at the First People’s Hospital of Foshan was performed. According to the Guidelines for the Diagnosis and Treatment of Allergic Rhinitis (2015 Edition), the recruit criteria were formulated: Patients with skin index (SI) of skin prick test (SPT) ++ or above, or specific Immunoglobulin E (sIgE) level in serum to dermatophagoides pteronyssinus (Der p) and/or dermatophagoides farinae (Der f) i. e., Derp/Derf≥0.35kU/L , which exposure to dust mites was confirmed as the major allergen by allergen tests, including: 1) Patients with mild to moderate asthma; 2) Patients with moderate to severe persistent rhinitis; 3) Mild to moderate asthma with allergic rhinitis (and/or allergic conjunctivitis); 4) Patients with mild to moderate asthma and eczema. Exclusion criteria included: 1) severe or uncontrolled bronchial asthma with continuous monitoring of Forced Expiratory Volume in one second (FEV1) <70% per of the expected value; 2) Patients with asthma whose symptoms or reduced lung function continue to fail to be controlled with grade 4 or 5 treatment; 3) Patients sensitized to other allergens such as pet furs, pollens or molds; 4) Patients who are taking beta-2 blockers or angiotensin-converting enzyme inhibitors; 5) Patients with serious underlying diseases, including cardiovascular and cerebrovascular diseases, autoimmune diseases and immunodeficiency diseases, malignant diseases, and chronic infectious diseases; 6) Patients with serious mental illness, lack of compliance, or inability to understand the risks and limitations of treatment. The patients data were anonymized before use.

The study protocol was approved by the Ethics Committee of the First People’s Hospital of Foshan, Foshan, China. All methods were performed in accordance with the relevant guidelines and regulations.

#### 2.1.2 SCIT treatment and evaluation

Before administering SCIT to enrolled patients, they will first perform a routine physical examination, inquire about related information since the last injection (including allergy symptoms), and post-injection, patients are observed for 30 min in case of the occurrence of side effects. Standardized adsorbed Der p and Der f allergen extracts (Allergopharma, Reinbeck, Germany) were used for SCIT. According to the manufacturer’s instructions, in the dose accumulation phase with weekly injections of allergen extracts with a gradually increased concentration from 100 SQ-U/mL to 10,000 SQ-U/mL, respectively injected 0.2, 0.4, 0.8 mL; after reaching the maintenance dose, 100,000 standardized quality units was used. In the maintenance phase, an injection interval of 6 ± 2 weeks was carried out according to the manufacturer’s recommendations.

Patients receive regular treatment evaluations, including symptom scores and medication scores. The symptom score recorded a total of nasal symptoms (nasal itching, sneezing, rhinorrhea, nasal congestion), ocular symptoms (ocular itching, lacrimation), and pulmonary symptoms (shortness of breath, tightness in chest, perennial cough, wheezing), and assessed symptom severity using the visual analogue scale (VAS). In the VAS symptom score, the score of each symptom is from 0 to 10.0 indicates that the patient has no discomfort and 10 indicates that the patient is extremely uncomfortable. The patient gives the score of each symptom according to the actual situation, and the sum of all symptom scores is the symptom score. Medication score recorded the use of current adjuvant medication within 1 month to reach symptom relief. The use of oral antihistamines, antileukotrienes, and bronchodilators were recorded as one point, local glucocorticoids as two points, oral glucocorticoids or combined medication (hormones and β2 receptor agonists) as three points, and the total cumulative score was the medication score. Symptom scores and medication scores were assessed once at registration of SCIT and then thereafter.

Due to the separated injection regimen within 16 weeks and thereafter, all the chosen patients completed the 4 months of SCIT, we chose the fourth month as the starting point of the observation. According to our previous experience, 1 year after the start was the peak of the withdrawal, so we added a time point at 18 months to further assess and follow up on the related symptom score and individual status. The data collection spans six time steps: at 0, 4, 12, 18, 24, and 36 months. This approach is standard in medical treatment, although for optimal model performance, an equal distribution of time intervals would be preferable.

#### 2.1.3 Data collection

Data were collected from patient records in hospitals, and the following information was extracted for analysis: patient age, gender, distance to clinic, ratio of AIT cost to family income, allergen test results, *etc.*, as well as patient VAS system score and medication score information, including baseline data of patients before injection therapy, adverse reactions to SCIT. For the descriptive analysis, categorical variables were given as numbers and percentages, and continuous variables were presented using mean, standard deviation, median, interquartile range (IQR), and minimum and maximum values. To address missing values, we tracked every patient, which allowed us to ensure the dataset’s completeness. We did not remove outliers, aiming to follow real-clinical scenarios as closely as possible.

#### 2.1.4 Survey methods

Adherence was defined as the accomplishment of 3 years of AIT. Non-adherence was defined as discontinuation of AIT at random time points during 3 years. The follow-up contents included 1) the main reasons for patients’ discontinuation of treatment; 2) the duration of discontinuation of treatment, and 3) Allergic symptoms after discontinuation of treatment.

### 2.2 Sequential models

The focus of our study is the development of sequential models that can efficiently and accurately predict the progression of symptoms and adherence in patients undergoing SCIT. This involves a comprehensive analysis of the data collected, structured to provide insights into the treatment’s effectiveness and patient compliance over time. Additionally, we explore and compare two distinct sequential models.

#### 2.2.1 Data

**FIGURE 2:**
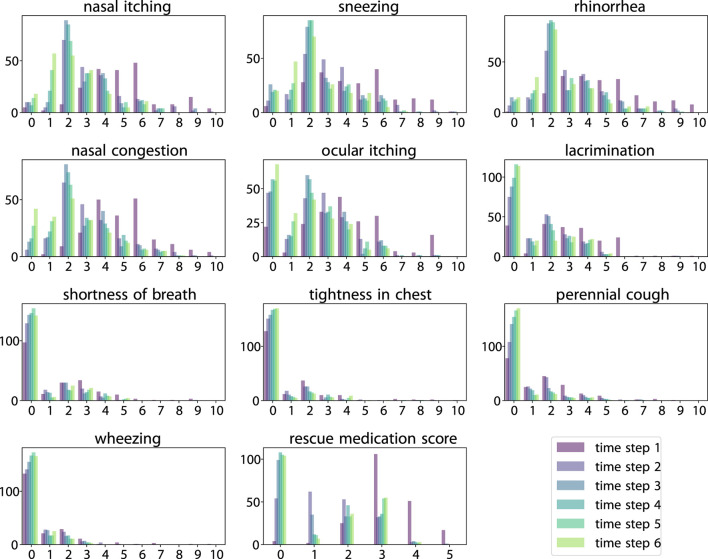
Histogram of scores across six time steps. Score value (horizontal axis) vs. count (vertical axis).

We have a dataset D , comprising sequences $x_{1},\ldots ,x_{T}\in R^{11}$ , $y_{1},\ldots ,y_{T-1}\in R^{1}$ , and a corresponding action $a_{t}\in R^{1}$ . In the context of healthcare, the observations encompass $y_{t}$ (see Table 2) whether the patient will cease the treatment in the interval between the scoring measurements at $x_{t}$ and $x_{t+1}$ . The actions $a_{t}$ represent the ongoing medical procedures for the patient during the period from $x_{t}$ to $x_{t+1}$ (see Figure 2). In this context, $a_{t}$ is binary, reflecting whether treatment is given, and is numerically equivalent to the adherence variable $y_{t}$ . Despite their numerical equivalence, we maintain a distinction between action $a_{t}$ and adherence $y_{t}$ to enhance model clarity and accommodate future research expansions, potentially allowing for a wider range of action values. For each patient, we possess basic information $s\in R^{14}$ which includes age, gender, commute distance to clinic, ratio of cost to family income, eosinophils count, eosinophils percentage, nasal allergen provocation test (change of nasal resistance, ΔNR(%) ), peak nasal inspiratory flow. ΔPNIF(%) ), serum total IgE level, sIgE of Dermatophagoides pteronyssinus (Derp), sIgE of Dermatophagoides farinae (Derf), skin prick test (Derp, Derf) (see Table 1 for more details).

**TABLE 1:** Demographic and clinical data of the patients under subcutaneous immunotherapy. In the rows from Age to Cost/Family income, values indicate the number of patients (percentage, if available). Other rows represent the median and IQR. *p*-values are omitted due to their large values.

Variables		Patients		
		total	adherent	non-adherent
age	≤12	96 (46.7)	40	56
	13–17	30 (14.6)	10	20
	≥18	79 (38.7)	23	56
gender	Female	62 (30.2)	22	40
	Male	143 (69.8)	51	92
distance to clinic (km)	≤10	136 (66.3)	56	80
	>10	69 (33.7)	17	52
cost/family income (%)	<30	107 (52.4)	37	70
	30–50	77 (37.4)	32	45
	>50	21 (10.2)	4	17
EOS( × $10^{9}$ /L)		0.37; 0.41	0.36; 0.52	0.38; 0.36
EOS %		0.05; 0.04	0.05; 0.05	0.05; 0.05
Δ NR (%)		16.67; 59.70	30.00; 92.80	14.80; 50.00
Δ PNIF(%)		11.90; 34.50	12.70; 39.30	11.10; 28.80
total IgE (kU/L)		286; 543	340; 487	226; 555
sIgE of Der p (kU/L)		30.80; 68.480	31.30; 74.40	30.40; 67.80
sIgE of Der f (kU/L)		40.00; 68.20	40.60; 75.10	37.10; 65.70
Der p SPT SI		1.04; 0.58	1.00; 0.59	0.82; 0.55
Der f SPT SI		1.00; 0.50	0.82; 0.51	0.80; 0.45

#### 2.2.2 Sequential latent variable model

In our research, we use the Stochastic Latent Actor-Critic (SLAC) model (Lee et al., 2020). Our application differs from the original use of SLAC which is typically associated with reinforcement learning. Instead, we use its sequential latent-variable model (SLVM) without Actor-Critic. This approach aligns with similar methodologies found in other works (Krishnan et al., 2015; Karl et al., 2016; Gregor et al., 2018). The choice of the SLAC model was motivated by its ability to facilitate more efficient learning and superior generalization in intricate environments.

The SLVM is fundamentally a framework that processes information in a step-by-step manner, capturing the dynamics of an environment or process over time. It constructs a latent representation of the data that it identifies and uses underlying patterns or structures within the dataset that probably are not immediately obvious. This capability makes it exceptionally suitable for tasks where understanding temporal relationships is crucial, such as predicting patient adherence in allergen immunotherapy. The model operates by generating a sequence of predictions, each informed by the data received up to that point, thereby enabling it to adapt and refine its understanding as more information becomes available. This methodological choice allows our research to use the strengths of SLAC in a novel context, applying it to the predictive modeling of patient behaviors in a healthcare setting.

After training the model, given the historical data up to step t−1 , the model is capable of generating a patient’s next scores from time step t to T directly from the latent space, i.e., $p\left(x_{t:T}|x_{0:t-1},a_{0:t-1}\right)$ . Similarly, it can predict the adherence of step t:T−1 at time step t , $p\left(y_{t:T-1}|x_{0:t},a_{0:t-1}\right)$ . See details of the model and the training process in Supplementary Appendix S1.

#### 2.2.3 LSTM

As an alternative, Long short-term memory (LSTM) is a classical sequential neural-network model (Hochreiter and Schmidhuber, 1997). Given the historical data, in our implementation, an LSTM predicts the score $x_{t+1}$ and adherence $y_{t}$ in parallel. The LSTM’s autoregressive feature allows us to iteratively input its current predictions to predict subsequent outcomes, covering prediction from $x_{t+1}$ to $x_{T}$ and $y_{t}$ to $y_{T-1}$ . Implementation details and the reasons for the model choice can be found in Supplementary Appendix S2.

## 3 Results

A total of 205 patients were enrolled in this study. The mean age was 17.57±11.68 years, children and adolescents represented the major population (61.3%) in AIT treatment. Males (70%) were predominantly represented. The population with a commute distance to the clinic within 10km was 66 percent. Due to a great portion of juveniles from the cohort, the ratio of cost to family income instead of personal income was evaluated. The patients who undertook AIT cost less than 30% of monthly family income and account for half the distribution of the population, while 12.9% non-adherent patients undertook the 50% financial burden in AIT treatment.

The change of nasal resistance (NR) and peak nasal inspiratory flow (PNIF) after nasal allergen provocation (NPT) was used to evaluate the severity of symptoms by combining the symptom score. The change of NR after NPT from the adherent group was higher than the non-adherent group (30% vs 14.8%) . The laboratory tests such as total IgE, sIgE of Der p and Der f, and SPT did not exhibit a significant difference between the two groups. For detailed characteristics of patients see Table 1.

The observed total non-adherence rate at the end of 3 years was 35.4% and the median of the SCIT duration was 18 months in the study. The rate of dropout in the third year (43.0%) was highest in comparison to the end of the first year (26.5%) and the second year (30.0%). The reason for the withdrawal from the patients included the concern of COVID-19, especially at the beginning of 2020 accounting for a 25% portion of the non-adherent patients in the first year. The most influential reason for the withdrawal was unreached expectations for clinical improvement (43.9%). Medical issues including pregnancy status during the treatment period and other physical disorders were collected from patients leading to the withdrawal of SCIT (4.5%). The significantly improved symptoms contributed to the reason for dropout, especially after 2 years SCIT treatments (18.2%). The recorded cases from side effects accounted for 4.5%, comprising the local and systematic adverse reactions (see Table 2).

**TABLE 2:** Detailed reasons for withdrawal from SCIT at different time points.

Reasons for SCIT withdrawal	Number of non-adherent patients				
	5–12 months	13–18 months	19–24 months	25–36 months	total by reason
no clinical improvement	18	11	8	21	58
medical issue	3	1	2	0	6
improved efficacy	0	0	0	24	24
schooling	3	3	0	5	11
side effects	2	1	1	2	6
COVID-19	9	7	3	1	20
personal issue	0	3	0	4	7
total by time period	35	26	14	57	132

We have a total of 205 samples, which we have randomly divided into a test dataset comprising 20%, i.e., 41 samples. For our analysis, we employ a five-fold cross-validation approach. Additionally, we apply zero-mean and unit standard deviation (STD) normalization to the variables x and s .

The Root Mean Square Error (RMSE) metric is used to evaluate the precision of our medical score predictions. Furthermore, to assess the adherence predictions, we use a comprehensive set of metrics including accuracy, precision, recall, and the F1 score, each offering a unique perspective on the performance of our predictive models. Most of the figures in this study are presented using boxplots.

In all results in this study, the uncertainties for both models are calculated using *five-fold cross-validation*. In addition, as SLAC is a probabilistic model, we also perform 100 samples from the latent space to compute its uncertainty.

### 3.1 One-step prediction

In this experiment, our focus is on predicting the immediate next step. Within the SLVM, the prediction of $y_{t}$ is based on the sequence $x_{1:t}$ and actions $a_{1:t-1}$ . Additionally, we forecast the subsequent state $x_{t+1}$ using the sequence $x_{1:t}$ along with actions $a_{1:t}$ . In contrast, for LSTM, the predictions for both $y_{t}$ and the next state $x_{t+1}$ are derived from $x_{1:t}$ and $y_{1:t-1}$ .

**FIGURE 3:**
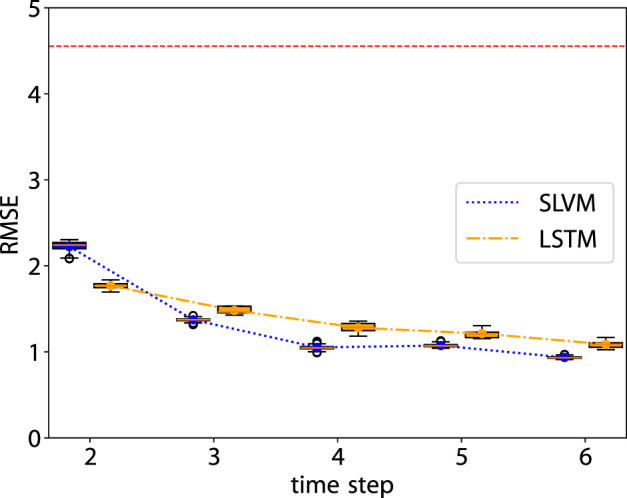
RMSE of the prediction step by step. The red dashed line is the RMSE of random prediction with Uniform distribution. See Figures 5, 6 for more details.

**FIGURE 4:**
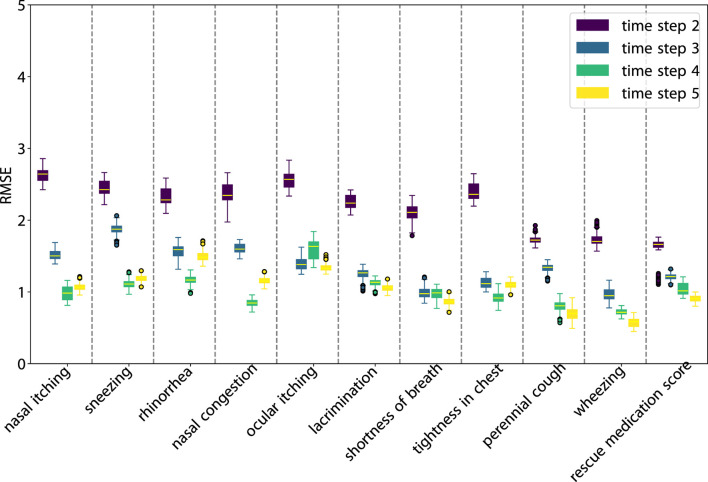
RMSE of SLAC one-step prediction across various scores and time steps.

**FIGURE 5:**
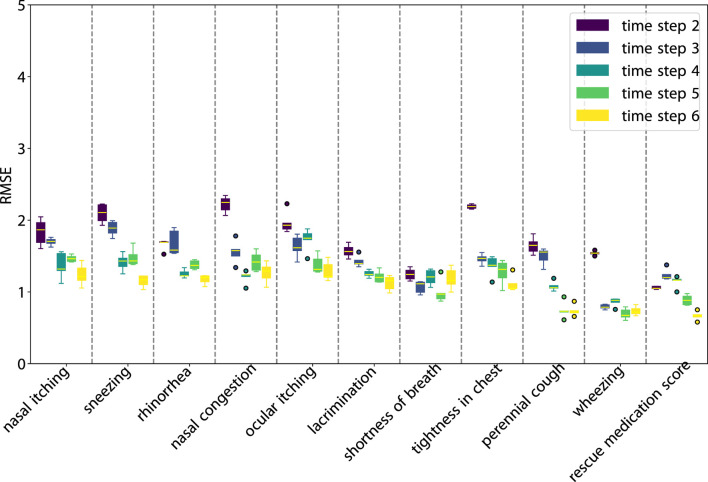
RMSE of LSTM one-step prediction across various scores and time steps.

As illustrated in Figure 3, SLVM surpasses LSTM in performance beginning at time step two. The figure indicates that with an increased amount of historical data (additional time steps), SLVM achieves greater RMSE. Both SLAC and LSTM demonstrate considerably better over random prediction methods. Further insights are provided in Figures 4, 5, which provides detailed representations of each feature. In the prediction of specific local symptoms score, SLVM performs an improvement in error after step two with all parameters compared to LSTM. The results from RMSE in nasal and ocular symptoms display relatively high values compared to those for lower respiratory tract symptoms. This can be attributed to the fact that the majority of patients in the cohort predominantly exhibited nasal and ocular symptoms, which presented a wide range of scores.

**FIGURE 6:**
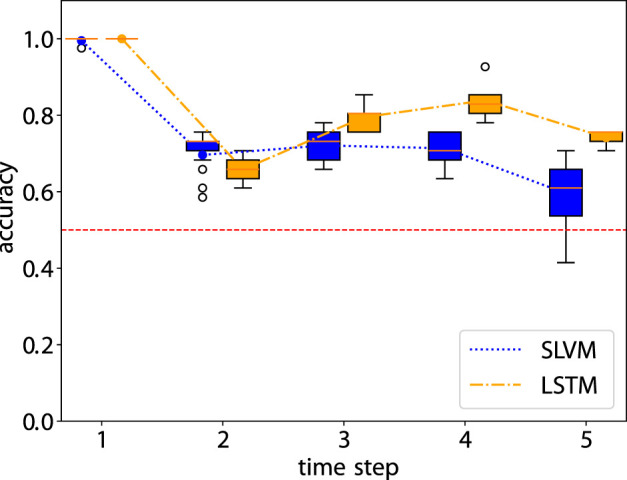
Accuray of the prediction step by step. The red dashed line is the accuracy of random prediction with Uniform distribution. See Table 3 for more details.

Figure 6 demonstrates that from steps two to four, accuracy in adherence predictions improves with the inclusion of additional information. The first step shows a notable bias, as it only includes data from adherent patients, as detailed in Sec. 2.1.2. Nonetheless, both models adeptly manage this bias and achieve high-accuracy predictions. Prediction for the sixth step is not conducted due to the cessation of treatment by the hospital. In the fifth step, there is a decline in accuracy, likely due to the extended time interval of 12 months. In future research, it would be worthwhile to explore whether adopting a consistent interval for data collection could enhance the outcomes of longitudinal prediction. Table 3 illustrates details of the classification for one-step prediction. Initially, both models exhibit perfect performance in Accuracy, Precision, and Recall at the first time step, but diverge in subsequent steps. In terms of Accuracy, LSTM generally outperforms SLVM, particularly evident at time steps three, four, and five. For Precision, LSTM again shows superior performance in the later time steps, except at time step two where SLVM marginally leads. However, in the Recall metric, SLVM surpasses LSTM from time step two onwards, indicating its strength in correctly identifying positive cases. The F1 score, which balances precision and recall, shows LSTM generally ahead, except at time step two where SLAC has a slight edge. This metric indicates LSTM’s balanced capability in both precision and recall, especially in the later time steps. Overall, while both models start equally strong, LSTM demonstrates greater consistency and effectiveness across most metrics and time steps. SLVM, while lagging slightly behind in accuracy and precision, shows its robustness in recall, especially in the middle to later time steps.

**TABLE 3:** Comparison of LSTM and SLAC over different time steps. The results are expressed as a mean ± standard deviation. The better results are highlighted in bold.

Metric	Model	Time step 1	Time step 2	Time step 3	Time step 4	Time step 5
accuracy	LSTM	1.00±0.00	0.66±0.03	0.80±0.04	0.84±0.05	0.74±0.02
	SLVM	1.00±0.01	0.70±0.06	0.72±0.04	0.71±0.04	0.60±0.06
precision	LSTM	1.00±0.00	0.72±0.01	0.86±0.06	0.90±0.05	0.62±0.03
	SLVM	1.00±0.00	0.75±0.03	0.74±0.03	0.71±0.03	0.44±0.05
recall	LSTM	1.00±0.00	0.86±0.06	0.83±0.05	0.82±0.08	0.61±0.03
	SLVM	1.00±0.01	0.87±0.06	0.90±0.03	0.86±0.04	0.70±0.10
F1 score	LSTM	1.00±0.00	0.79±0.03	0.84±0.03	0.85±0.05	0.62±0.03
	SLVM	1.00±0.00	0.81±0.04	0.81±0.02	0.78±0.03	0.54±0.06

### 3.2 Rollouts

In the rollout experiment, our focus extends to a longer-term prediction. The SLAC prediction of $y_{t:T-1}$ and $x_{t+1:T}$ are computed based on $x_{1:t}$ and $a_{1:t-1}$ . Actions, $\left\{a_{i}:i\geq t\right\}$ are inferred from the model’s output, $y_{t}$ . Moreover, for time steps greater than t , we employ a prior in the latent space, which eliminates the need for the input of $x_{t:T}$ . In the LSTM model, the predictions for $y_{t:T-1}$ and $x_{t+1:T}$ are based on $x_{1:t}$ and $y_{1:t-1}$ .

**FIGURE 7:**
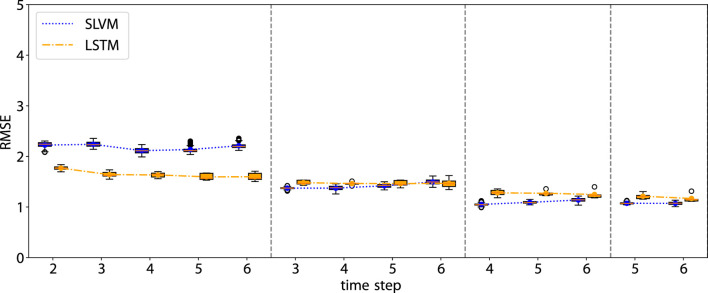
RMSE of the rollout prediction. The first time step in each subplot represents the beginning of the rollout time step.

**FIGURE 8:**
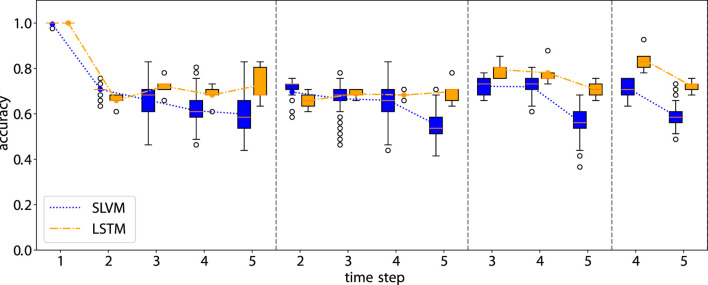
Accuracy of the rollout prediction. The first time step in each subplot represents the beginning of the rollout time step.

Figures 7, 8 illustrate the performance of our model in multi-step predictions. Similar to one-step predictions, the accuracy generally improves with the availability of more information, except in the case of the adherence prediction at the fifth step. The results demonstrate the model’s proficiency in making long-term predictions.

### 3.3 Model as a simulator

Given the initial condition of a patient, we can assess the outcomes of various interventions. Clinically, if the patient’s adherence to treatment significantly impacts the prognosis (and there is a possibility of non-adherence), it becomes imperative for the doctor to emphasize treatment compliance. Conversely, if adherence makes little difference, it suggests the therapeutic approach may be ineffective for this patient, allowing the doctors to emphasize adherence efforts.

To evaluate the impact of varying actions on SLAC’s performance, we analyze how different actions affect the resulting scores. In the absence of a ground truth with diverse actions for the same patient, our focus shifts to examining whether the states are responsive to changes in actions. Considering initial states $x_{1:3}$ and actions $a_{1:2}$ , we do rollouts with $a_{3:5}$ , alternating between one and zero. This controlled alteration reveals that the average predicted value of $x_{6}$ under these conditions is −0.20 . This value is computed from the prediction outcomes for actions with ones minus those for actions with zeros. The result indicates that our model can be used as a simulator for doctors to see the impact of different treatments/therapies. Since the LSTM does not have similar functions (see Section 2.2.3), we only show the SLAC results.

### 3.4 Interpretability

**FIGURE 9:**
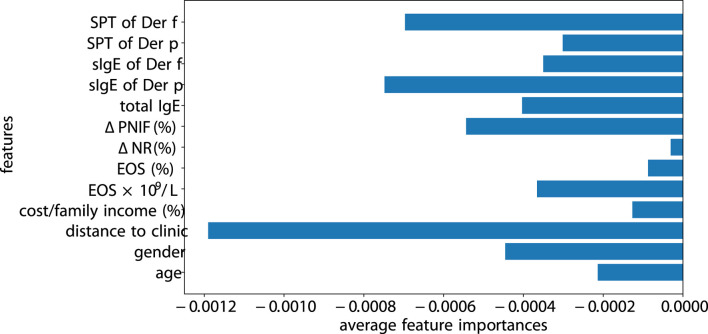
Importances of the factors.

Previous models and methods have been developed for interpreting machine learning algorithms, including SHAP (Lundberg and Lee, 2017) and Captum (Kokhlikyan et al., 2020). We opt for Captum, as it integrates more seamlessly with PyTorch-based code. We perform the measure of the factor importance using Integrated Gradients of Captum for SLAC (see Figure 9). The magnitude of features highlights the significance of the model’s prediction for a specific class. The distance to the clinic significantly impacts patient adherence, especially if a patient is located far from the clinic or has relocated, as they are more likely to discontinue their visits. Following the distance, SPT of Der f and sIgE of Der f greatly influence the adherence. In contrast, ΔNR(%) , EOS (%) , and the cost/family income (%) have minimal impacts.

## 4 Discussion and conclusion

The reported adherence rates of SCIT ranged from around 23%–90%, due to the non-uniform follow-up duration (2–4 years) (Passalacqua et al., 2013; Lemberg et al., 2017; Yang et al., 2018; Lee et al., 2019). Poor adherence in the three to 5-year time span of AIT is an obstacle to reaching allergen tolerance and symptom remission. Recently “adherence and persistence in AIT (APAIT)” checklist was proposed to assists researchers in assessing adherence or persistence to AIT treatment (Pfaar et al., 2023). The present study is the first research regarding the application of machine learning models in the adherence prediction of SCIT in AR patients. From our study, the accomplishment rate of the 3-year treatment cycle was relatively low (35.4%), while the dropout rate after 2 years accounts for half (42.8%) in the whole non-adherence cohort. Several researchers focus on these variables impacting adherence to medical behaviors to enhance the intervention approach to reduce the withdrawal caused by disease-unrelated reasons. Even though the COVID-19 pandemic affected the adherence of the majority of the patients in the 3-year cycle, the first year since the pandemic’s outbreak appears to have fundamentally built a barrier to patients, similar to the finding from Liu et al. (2021) that 11% dropouts in the 2 years SCIT was observed caused by COVID-19. We excluded the patients who dropped out in the dose buildup phase within 4 months to minimize the dose-origin impact. Due to the uncovered cost from the public healthcare system and commercial insurance, financial burden accounts for a non-negligible factor in influencing the patient’s decision-making. A similar finding from Lourenço et al. (2020) indicated that economic reasons contributed to the most frequent cause of SCIT cessation.

Recently, as the presented studies focusing on the medication adherence prediction of non-communicable diseases such as diabetes, hypertension, cancer, and chronic respiratory diseases regarding machine learning models were introduced into the application, the systematic monitoring of patients’ adherence behaviors remarkably re-tailored the disease management and enhanced medical decision-making (Kanyongo and Ezugwu, 2023) Due to the multidimensional variables in the prediction of adherence collected for analysis, machine learning models exhibit advantages in automatic feature selection, interaction effects, scalability, robustness, and so on compared to traditional regression analysis.

Mousavi et al. (2022) demonstrated the effectiveness of a hybrid model that combines neural networks and genetic algorithms for predicting diet adherence. Wang et al. (2020) explored another hybrid model that integrates neural networks and support vector machines to predict nonadherence in Crohn’s Disease patients by streamlining the intervention process in medicine-taking. Both methods have shown excellent performance. The deployment of machine-learning algorithms in the prediction of adherence in cardiovascular disease including random forests, support vector machines, and neural networks showed the accuracy ranged from 0.53 to 0.97 (Mirzadeh et al., 2022; Zakeri et al., 2022). The ensemble learning model in the prediction of adherence from the patients who conducted self-administer injections proposed by Gu et al. (2021) achieved a good performance and generalization properties based on the fusion of multiple heterogeneous classifiers. In the field of allergen immunotherapy, Yao et al. (2023) introduced a machine-learning model with an improved DFSSA algorithm to predict the therapeutic efficacy of AIT for asthma using clinical characteristics and serum allergen detection metrics. However, these non-sequential methods generally predict only the final outcome, neglecting the complexities of intermediate stages. A sequential model that can make predictions at any specific time step would significantly enhance the ability for early intervention. Hsu et al. (2022) investigated the advantages of incorporating patient history into the prediction of medication adherence. They assessed the performance of temporal neural network models, particularly LSTM and simple recurrent neural networks, and compared these with non-temporal neural networks, ridge classifiers, and logistic regression. To optimize the efficacy of cognitive training for older adults, Singh et al. (2022) employed multivariate time series analysis and developed personalized models for each patient to capture their unique adherence patterns. However, the sequential data of patients is often characterized by fluctuating adherence and high dropout rates, resulting in uneven, unaligned, and missing values in the time series data. To address this challenge, Schleicher et al. (2023) applied change point detection to identify phases with varying dropout rates, presented methods for handling uneven and misaligned time series, and used time series classification to predict the user’s phase. These models, however, overlook the significance of score prediction in SCIT treatment. Our study advances this methodology by integrating a state-action model capable of predicting both adherence and score/state. This enhancement facilitates a more accurate and comprehensive whole-process management of AR patients in SCIT treatment.

The proposed prediction models can help clinicians dynamically measure the effectiveness of adherence interventions including more frequent reminders or engagement strategies, such that healthcare teams can focus on these individuals and proactively provide them with additional support. Moreover, in the surveillance of local symptom scores and rescue medicine up-take, the models offer a clinical evidence-based approach to precisely predict the risk of non-adherence in patient-centered care precisely. Especially for the potential risk of withdrawal caused by medical issues or related side effects, our models suggest multidimensional observational parameters for timely offering medical intervention and increasing patient engagement by participating in shared decision-making. The implementation of integration of the models with existing healthcare workflows is challenging, while the application of telesystem and online consultation would improve the work efficacy in immunotherapy centers and facilitate patient’s self-management.

Our study demonstrates notable findings in the domain of patient adherence prediction in subcutaneous immunotherapy. The comparison between the SLAC model and LSTM model reveals the distinct strengths and limitations of each approach. Notably, SLAC exhibits greater flexibility, and it outperforms LSTM in score prediction. This advantage likely stems from its ability to efficiently learn and generalize in complex environments. Conversely, the LSTM model shows better performance in predicting adherence, indicating its potential usage in scenarios. Both models demonstrate the capability to handle longer sequences, extending beyond one-step prediction. This ability is crucial in medical settings where long-term patient monitoring and prediction are essential for effective treatment planning.

Overall, the study underscores the importance of selecting the appropriate model based on the specific requirements of the task, whether it be flexibility, precision in score prediction, or adherence prediction. The findings contribute to the growing field of machine learning applications in healthcare, particularly in enhancing patient-centered treatment strategies through accurate and personalized predictions. Future research could focus on evaluating the SLAC model’s performance in simulating various actions, further enriching its applicability in clinical settings. Additionally, the generalization to other diseases or the application of our models would be an interesting direction for future research.

## Data Availability

The original contributions presented in the study are included in the article/Supplementary Material, further inquiries can be directed to the corresponding author.
