# Supplementary material for: Sequential model for predicting patient adherence in subcutaneous immunotherapy for allergic rhinitis
Source: Front Pharmacol. 2024 Jul 19;15:1371504. doi: 10.3389/fphar.2024.1371504 (PMC11294208; doi:10.3389/fphar.2024.1371504)
Supplement: Supplementary file 1 [file Presentation1.pdf]

Supplementary Material for Sequential Model for Predicting Patient Adherence in Subcutaneous Immunotherapy for Allergic Rhinitis

Yin Li [1,*], Yu Xiong [2*], Wenxin Fan [3], Kai Wang [1], Qingqing Yu [1], Liping Si [4], Patrick van der Smagt [5,6], Jun Tang [1⋆] and Nutan Chen [6]

[1] *Department of Otorhinolaryngology, The First People's Hospital of Foshan, China*
[2] *Department of Otorhinolaryngology, The Second Affiliated Hospital of Guizhou University of Traditional Chinese Medicine, Guiyang, China*
[3] *Paul C. Lauterbur Research Center for Biomedical Imaging, Shenzhen Institutes of Advanced Technology, Shenzhen, China*
[4] *Department of Radiology, Zhongshan Hospital, Fudan University, Shanghai, China*
[5] *Faculty of Informatics, ELTE University, Budapest, Hungary*
[6] *Machine Learning Research Lab, Volkswagen Group, Munich, Germany*

Correspondence*:
Jun Tang
fsyyytj@126.com

1 LATENT SEQUENTIAL VARIABLE MODEL

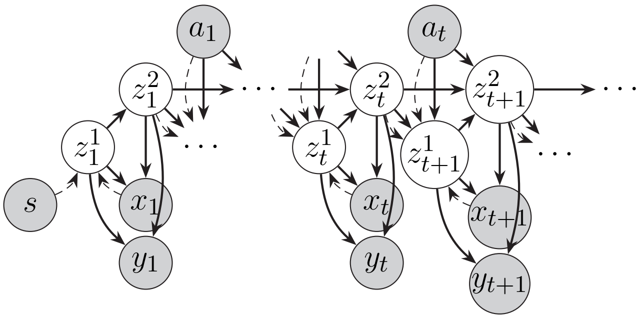

Figure 1. Schematic of the LSVM part of SLAC. Solid and dashed lines denote the generative and inference model pathways, respectively. The gray circles represent observed data, and the white circles denote latent variables. The figure is adapted from (Lee et al., 2020).

The sequential latent variable model (SLVM) of the SLAC consists of an inference model and a generative model (see Fig. 1). The inference model in a sequential latent-variable model typically aims to approximate the posterior distribution of the latent variables given the observed data. It tries to infer the hidden states z

[*] These authors contributed equally to this work.

based on the observed inputs x and initial states s. The inference models the probability distributions of the latent variables z^1 and z^2 at different time steps. q_ϕ denotes the variational distribution parameterized by ϕ,

$$z_1^1 \sim q_\phi(z_1^1 \mid x_1, s) \tag{1}$$
$$z_1^2 \sim p_\phi(z_1^2 \mid z_1^1) \tag{2}$$
$$z_{t+1}^1 \sim q_\phi(z_{t+1}^1 \mid x_{t+1}, z_t^2, a_t) \tag{3}$$
$$z_{t+1}^2 \sim p_\phi(z_{t+1}^2 \mid z_{t+1}^1, z_t^2, a_t). \tag{4}$$

The generative model, on the other hand, describes how the observed data is generated from the latent variables. The generative model is the probability distribution of both the initial latent states and their transitions over time, as well as the likelihood of the observations given the latent states, with p_ϕ indicating the parameterized generative distribution.

$$z_1^1 \sim p(z_1^1) \tag{5}$$
$$z_1^2 \sim p_\phi(z_1^2 \mid z_1^1) \tag{6}$$
$$z_{t+1}^1 \sim p_\phi(z_{t+1}^1 \mid z_t^2, a_t) \tag{7}$$
$$z_{t+1}^2 \sim p_\phi(z_{t+1}^2 \mid z_{t+1}^1, z_t^2, a_t) \tag{8}$$
$$x_t \sim p_\phi(x_t \mid z_t^1, z_t^2) \tag{9}$$
$$y_t \sim p_\phi(y_t \mid z_t^1, z_t^2). \tag{10}$$

We have the evidence lower bound (ELBO):

$$\log p_\phi(x_{1:t+1}|a_{1:t}) \geq \Bigg[\mathop{\mathbb{E}}_{(x_{1:T}, a_{1:T-1}) \sim D} \Bigg[\mathop{\mathbb{E}}_{z_{1:T} \sim q_\phi} \sum_{t=0}^{T-1} \Big(\log p_\phi(x_{t+1} \mid z_{t+1}) - D_{KL}\big(q_\phi(z_{t+1} \mid x_{t+1}, z_t, a_t) \parallel p_\phi(z_{t+1} \mid z_t, a_t)\big)\Big)\Bigg]\Bigg]. \tag{11}$$

For ease of notation, we have $q(z_1 \mid x_1, z_0, a_0) := q(z_1 \mid x_1, s)$ and $p(z_1 \mid z_0, a_0) := p(z_1)$. The ELBO provides a lower bound to the log-likelihood of the observed data, which is computationally intractable to compute directly. It is composed of two terms: the expected log-likelihood of the observed data given the latent variables, and the Kullback-Leibler (KL) divergence between the variational distribution and the prior distribution of the latent variables. Minimizing the KL divergence can be interpreted as enforcing the variational distribution to be as close as possible to the prior, while maximizing the expected log-likelihood ensures that the model accurately captures the distribution of the observed data. To predict the adherence, we have $\log p_\phi(y_{t+1}|z_{t+1})$ as a regularisor in the loss function.

The objective is to compute the parameters ϕ that minimize the KL divergence between the variational and prior distributions of the latent variables, subject to certain constraints. These constraints are related to the expected log-likelihood of the data under the model and are represented by the inequalities with thresholds ξ. These thresholds ensure that while minimizing the losses, the model also satisfies a minimum standard for score prediction and adherence classification performances.

Latent variable models, such as Variational Autoencoders (VAEs) (Kingma and Welling, 2014; Rezende et al., 2014) and their variants (e.g., SLAC), often encounter challenges (Sønderby et al., 2016; Kingma et al., 2016). Furthermore, a higher ELBO does not always lead to enhanced predictive performance, as discussed by Alemi et al. (2018); Higgins et al. (2017). However, the integration of scheduling strategies inspired by constrained optimization methods has been shown to significantly improve the training of latent variable models (Rezende and Viola, 2018; Klushyn et al., 2019; Sun et al., 2024). Consequently, we formulate the training of our model into an optimization problem

$$\min_{\phi} \mathop{\mathbb{E}}_{(x_{1:T}, a_{1:T-1}) \sim D} \left[\sum_{t=0}^{T-1} \left[D_{KL} \left(q_\phi(z_{t+1} \mid x_{t+1}, z_t, a_t) \parallel p_\phi(z_{t+1} \mid z_t, a_t) \right) \right] \right] \tag{12}$$

$$\text{s.t.} \quad \mathop{\mathbb{E}}_{(x_{1:T}, a_{1:T-1}) \sim D} \left[\mathop{\mathbb{E}}_{z_{1:T} \sim q_\phi} \left[\sum_{t=0}^{T-1} - \log p_\phi(x_{t+1} \mid z_{t+1}) \right] \right] \leq \xi_{\text{score}} \tag{13}$$

$$\mathop{\mathbb{E}}_{(x_{1:T}, a_{1:T-1}, y_{1:T-1}) \sim D} \left[\mathop{\mathbb{E}}_{z_{1:T} \sim q_\phi} \left[\sum_{t=0}^{T-2} - \log p_\phi(y_{t+1} \mid z_{t+1}) \right] \right] \leq \xi_{\text{adherence}} \tag{14}$$

where ξ is a baseline error, in Eq. (13) we have regression with Gaussian distribution, and in Eq. (14) we use cross-binary entropy loss for classification. To solve the optimization problem, we incorporate the constraints into the objective function using Lagrange multipliers λ. We apply methods from (Chen et al., 2022) to adapt λ. This allows the model to balance the importance of the constraints relative to the divergence terms, which can help in avoiding common pitfalls in training such as suboptimal local minima and posterior collapse.

To avoid over-fitting, we incorporate dropout (Srivastava et al., 2014) and Mixup (Zhang et al., 2017). Subsequent research has extended the application of Mixup to latent variable models, specifically within the latent space (e.g., (Chen et al., 2020)). However, considering our need for data augmentation across all data dimensions, not limited to latent variables, we have selected to implement the original Mixup method in our experiments.

2 LSTM

The primary objective of this study is to forecast y_t from historical data, formulated as $y_t = f(x_{1:t}, y_{1:t-1}, s)$. To align this approach with the SLVM of SLAC for score prediction, an additional term x_{t+1} is also predicted,

$$(x_{t+1}, y_t) = f(x_{1:t}, y_{1:t-1}, s) \tag{15}$$

where f is a function represented by an LSTM. The loss consists of the cross entropy for adherence classification and the Normalized Mean Squared Error Loss (NMSE) for score prediction.

In our scenarios, SLVM stands out due to its inherent flexibility over traditional sequential models like LSTM. This flexibility is primarily observed in its predictive capabilities. SLVM can predict y_t and use this prediction to influence the subsequent x_{t+1}. In contrast, LSTM only predicts a pair of y_t and x_{t+1} simultaneously, implying that we cannot use y_t to alter x_{t+1}. Although it is possible to modify the LSTM model to predict a pair of y_t and x_t, this approach encounters a similar issue for y_t: it cannot predict y_t using the information from x_t.

3 ARCHITECTURE AND COMPUTATION

In this study, computational experiments were performed using an NVIDIA GeForce GTX 1080 Ti GPU, with the implementation done in PyTorch, version 2.1.0.

The SLVM model's architecture featured 32 hidden dimensions each for variables z_1 and z_2. Its encoder and decoder were symmetrically structured, each comprising five layers with 128 units. The primary activation function was LeakyReLU, set with a negative slope coefficient of 0.2. Both the encoder and decoder's mean output layers were linear, while the STD layer utilized a Softplus activation. For binary classification tasks, a Sigmoid activation was used for output.

The LSTM architecture included a hidden dimension size of 128, with two LSTM layers. The output activation function for score prediction was linear, and as in the SLVM model, a Sigmoid function was used for binary classification outputs.

Both models shared the same optimization settings. They used the RAdam (Liu et al., 2019) optimizer with a learning rate of 0.001. The batch size was set at 64, and a gradient clipping value of 0.8 was applied to ensure training stability. To prevent overfitting and enhance model generalization, a dropout rate of 0.05 was introduced. Additionally, both models incorporated Mixup as a data augmentation during training.

REFERENCES

Alemi, A. A., Poole, B., Fischer, I., Dillon, J. V., Saurous, R. A., and Murphy, K. (2018). Fixing a broken ELBO. *ICML*

Chen, N., Klushyn, A., Ferroni, F., Bayer, J., and Van Der Smagt, P. (2020). Learning flat latent manifolds with vaes. *ICML*

Chen, N., van der Smagt, P., and Cseke, B. (2022). Local distance preserving auto-encoders using continuous knn graphs. In *Topological, Algebraic and Geometric Learning Workshops 2022*. 55–66

Higgins, I., Matthey, L., Pal, A., Burgess, C., Glorot, X., Botvinick, M., et al. (2017). Beta-VAE: Learning basic visual concepts with a constrained variational framework. *ICLR*

Kingma, D. P., Salimans, T., Jozefowicz, R., Chen, X., Sutskever, I., and Welling, M. (2016). Improved variational inference with inverse autoregressive flow. *Advances in neural information processing systems* 29

Kingma, D. P. and Welling, M. (2014). Auto-encoding variational Bayes. *ICML*

Klushyn, A., Chen, N., Kurle, R., Cseke, B., and van der Smagt, P. (2019). Learning hierarchical priors in VAEs. *Advances in Neural Information processing Systems* 32

Lee, A. X., Nagabandi, A., Abbeel, P., and Levine, S. (2020). Stochastic latent actor-critic: Deep reinforcement learning with a latent variable model. *Advances in Neural Information Processing Systems* 33, 741–752

Liu, L., Jiang, H., He, P., Chen, W., Liu, X., Gao, J., et al. (2019). On the variance of the adaptive learning rate and beyond. *arXiv preprint arXiv:1908.03265*

Rezende, D. J., Mohamed, S., and Wierstra, D. (2014). Stochastic backpropagation and approximate inference in deep generative models. In *ICML*. vol. 32, 1278–1286

Rezende, D. J. and Viola, F. (2018). Taming VAEs. *CoRR*

Sønderby, T., C. K.and Raiko, Maaløe, L., Sønderby, S. K., and Winther, O. (2016). Ladder variational autoencoders. *NeurIPS*

Srivastava, N., Hinton, G., Krizhevsky, A., Sutskever, I., and Salakhutdinov, R. (2014). Dropout: a simple way to prevent neural networks from overfitting. *The journal of machine learning research* 15, 1929–1958

Sun, X., Chen, N., Gossmann, A., Xing, Y., Feistner, C., Dorigatt, E., et al. (2024). M-hof-opt: Multi-objective hierarchical output feedback optimization via multiplier induced loss landscape scheduling. *arXiv preprint arXiv:2403.13728*

Zhang, H., Cisse, M., Dauphin, Y. N., and Lopez-Paz, D. (2017). mixup: Beyond empirical risk minimization. *arXiv preprint arXiv:1710.09412*
